# The Role of Peripheral Opioid Receptors in Triggering Heroin-induced Brain Hypoxia

**DOI:** 10.1038/s41598-020-57768-3

**Published:** 2020-01-21

**Authors:** David Perekopskiy, Anum Afzal, Shelley N. Jackson, Ludovic Muller, Amina S. Woods, Eugene A. Kiyatkin

**Affiliations:** Behavioral Neuroscience Branch, National Institute on Drug Abuse – Intramural Research Program, National Institutes of Health, DHHS, 333 Cassell Drive, Baltimore, MD 21224 USA

**Keywords:** Preclinical research, Neurophysiology

## Abstract

While it is known that opioid receptors (ORs) are densely expressed in both the brain and periphery, it is widely accepted that hypoxic effects of opioids result solely from their direct action in the CNS. To examine the role of peripheral ORs in triggering brain hypoxia, we used oxygen sensors in freely moving rats to examine how naloxone-HCl and naloxone-methiodide, the latter which is commonly believed to be peripherally restricted, affect brain oxygen responses induced by intravenous heroin at low, human-relevant doses. Similar to naloxone-HCl, naloxone-methiodide at a relatively low dose (2 mg/kg) fully blocked heroin-induced decreases in brain oxygen levels. As measured by mass spectrometry, naloxone-methiodide was found to be ~40-fold less permeable than naloxone-HCl across the blood-brain barrier, thus acting as a selective blocker of peripheral ORs. Despite this selectivity, a low but detectable amount of naloxone was found in brain tissue after naloxone-methiodide administration, potentially influencing our results. Therefore, we examined the effects of naloxone-methiodide at a very low dose (0.2 mg/kg; at which naloxone was undetectable in brain tissue) and found that this drug still powerfully attenuates heroin-induced brain oxygen responses. These data demonstrate the role of peripheral ORs in triggering heroin-induced respiratory depression and subsequent brain hypoxia.

## Introduction

Respiratory depression that leads to brain hypoxia appears to be the most dangerous effect of opioid drugs^1–5^. While this side-effect of opioids is minor following their therapeutic use, it is a leading cause of coma and death when highly potent opioid drugs such as heroin or fentanyl are self-administered at high doses via routes that provide rapid drug delivery to the CNS.

It is generally believed that respiratory depression results from the direct interaction of opioid drugs with opioid (mainly μ) receptors (ORs) expressed on brainstem neurons of the breathing center as well as other central neurons^6–9^. This basic mechanism has solid experimental support: respiratory activity has been shown to rapidly decrease following intracerebral, intercisternal, and local intra-brain microinjections of various opioid agonists^10–12^. This suggests an important role for centrally located ORs in descending mechanisms regulating respiratory activity. However, ORs are also abundantly expressed in afferents of sensory nerves innervating blood vessels, skin and internal organs, including lung airways^13,14^. Therefore, in addition to central targets, systemically administered opioid drugs directly interact with peripherally located ORs and change afferent inputs to the CNS, thus inducing neural effects and leading to subsequent changes in physiological parameters.

Since opioid drugs interact with both centrally and peripherally located ORs, comparing the effects of opioid antagonists which cross the blood-brain barrier (BBB) with ones that are unable to cross this barrier could be an effective tool for examining the role of peripheral receptors in triggering the neural and physiological effects of opioids. Naloxone-HCl, a highly potent non-selective opioid antagonist that easily crosses the BBB and interacts with both centrally and peripherally located ORs, fully blocks respiratory depression as well as other physiological and behavioral effects of morphine, heroin and fentanyl^7^. However, respiratory depression induced by morphine and fentanyl is also attenuated by large-dose naloxone-methiodide (naloxone-MET^15,16^), a quaternary analog of naloxone which is believed to be impermeable to the BBB and thus would interact only with the peripheral pool of ORs^16–20^. Naloxone-MET is also able to affect nociceptive and gastro-intestinal responses induced by morphine^16^, fentanyl^15^, and oxycodone^21^.

The primary goal of this study was to evaluate the role of peripheral ORs in mediating brain hypoxia induced by intravenous (iv) heroin in awake, freely-moving rats. The use of oxygen sensors coupled with amperometry allowed us to directly examine drug-induced changes in brain oxygen levels—a functional and integrative output of respiratory activity. While respiratory activity is the primary means for oxygen delivery into brain tissue, oxygen dissolved in the brain’s extracellular space is a more clinically relevant parameter and its decrease below physiological range is the direct cause of death during opioid overdose. Another critical advantage of this technology is its second-scale temporal resolution that allows for the observation of rapid drug-induced oxygen changes under physiologically relevant conditions. As shown previously, brain oxygen levels rapidly increase following exposure to various sensory stimuli^22^ and dose-dependently decrease following administration of various opioid drugs^23^. As in our previous studies, oxygen recordings were conducted in the nucleus accumbens (NAc), a deep, ventrally located structure critically involved in sensorimotor integration and drug reinforcement^24,25^.

This study followed a three-part progression, in which each subsequent experiment was designed to answer questions arising from the previous experiment. Initially, we examined how the effects of iv heroin would be affected by naloxone-HCl and naloxone-MET, assuming as other researchers that naloxone-MET does not enter brain tissue^16–20^. Heroin was delivered at a low, physiologically relevant dose (0.1 mg/kg) that is optimal for maintaining iv self-administration^26^; heroin at this dose induces moderate brain hypoxia^27^. In contrast to previous studies, in which large doses of opioid antagonists were often used, the dose of naloxone-HCl was relatively small (0.2 mg/kg). Naloxone-MET was delivered at 2.0 mg/kg to compensate for its 10–15-fold lower affinity for μ-opioid receptors^28–30^. To clarify the mechanisms underlying heroin-induced NAc oxygen responses and their changes following administration of opioid antagonists we conducted two additional experiments, using the same experimental protocol but monitoring different parameters. In the first experiment, we conducted oxygen recordings in the subcutaneous space, a densely vascularized location with no (or minimal) metabolic activity of its own, thus a valuable proxy of blood oxygen levels, which directly reflect the efficiency of respiratory activity^31^. In the second experiment, we examined drug-induced changes in locomotion and temperature in the NAc, temporal muscle, and skin. This three-point temperature-recording paradigm makes it possible to evaluate the effects of drugs on brain metabolic activity and peripheral vascular tone^32^.

Although it is generally accepted that naloxone-MET does not permeate the BBB (see above), we found a lack of objective proof to support this claim. To make sure that our results were not influenced by possible entry of naloxone-MET into brain tissue, in the second stage of the study we used liquid chromatography-mass spectrometry (LC-MS) to examine the extent to which both naloxone analogs, when delivered systemically, permeate the brain.

Finally, our third stage of the study was conducted in response to our LC-MS findings. In this oxygen experiment we examined whether and how the effects of iv heroin would be affected by systemic administration of naloxone-MET at a lower dose (0.24 mg/kg), the molar equivalent of the low dose of naloxone-HCl used in our experiments. At this dose, no reliable amounts of methyl-naloxone, the *in vivo* form of naloxone-MET, were found in brain tissue.

## Results

### Heroin-induced changes in brain oxygen, brain temperature, and locomotion

For proper assessment of the effects of opioid antagonists on heroin-induced responses, it is important to examine these responses and their underlying mechanisms in intact rats preceding the use of antagonists. Therefore, at the first stage of our analysis, we examined changes in oxygen levels in the NAc and subcutaneous space, temperature parameters, and locomotion induced by iv heroin in intact, drug-free rats.

**Figure 1:**
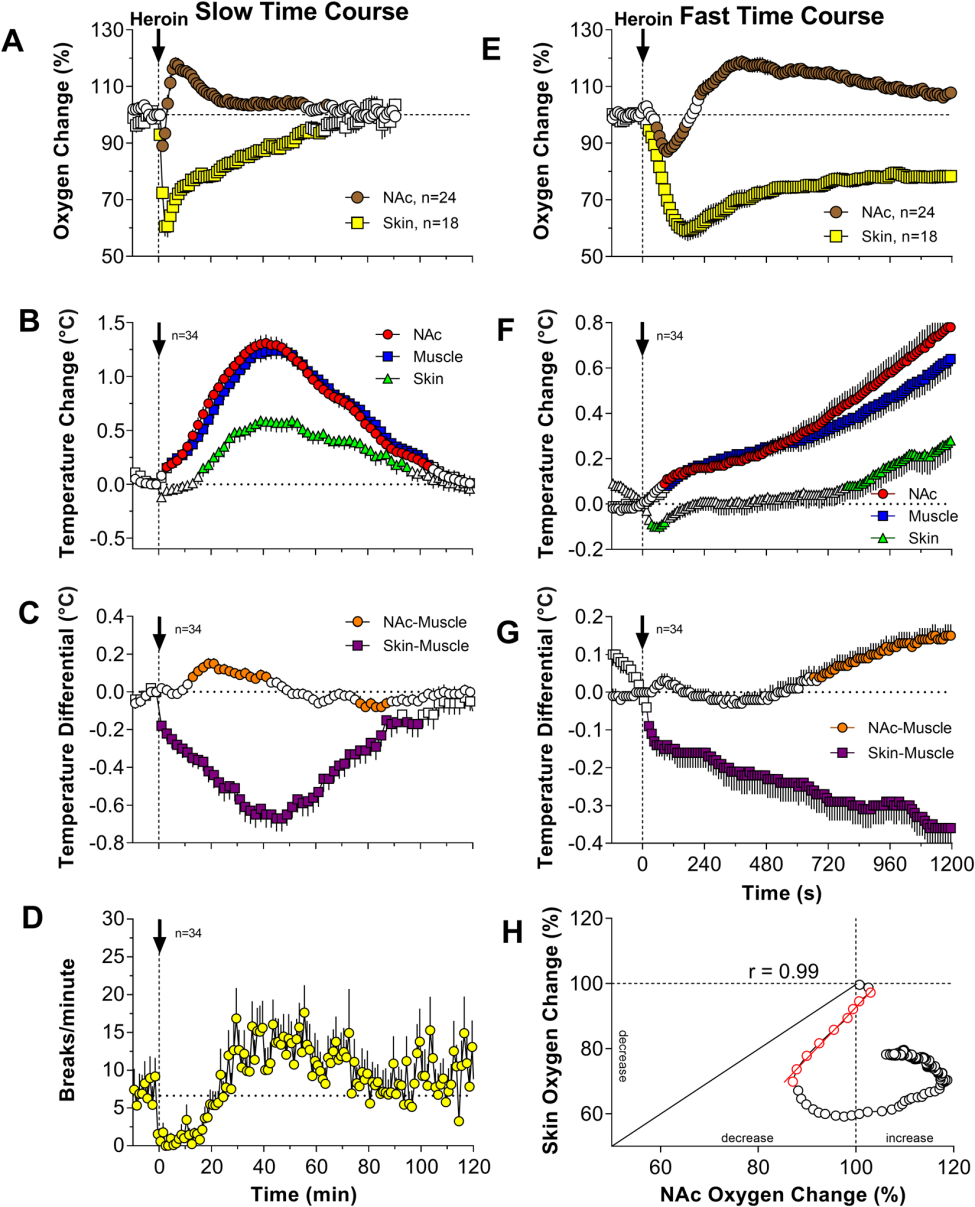
Mean (±SEM) changes in NAc and subcutaneous oxygen levels, temperature parameters, and locomotion induced by iv heroin delivered to awake, quietly resting rats at a low, self-administering dose (0.1 mg/kg). Left panels (A–D) show changes assessed with slow time-resolution (1-min bins) and right panels (E–G) show changes assessed at high temporal resolution (10-s bins). Values significantly different from baseline are shown as filled symbols; n indicates the number of averaged tests. Vertical dotted lines show the moment of iv heroin injection and horizontal dotted lines show baselines. H shows the correlation between oxygen changes in the NAc and subcutaneous space for the first 20 min from the onset of the injection. Red symbols show values where both parameters tightly correlated (red line is the regression line).

When analyzed with slow, 1-min time resolution, iv heroin induced a biphasic effect on NAc oxygen levels (F_23,2093_ = 16.51, p < 0.0001; Fig. 1A). Oxygen levels rapidly decreased immediately after the injection, reached nadir at 2–3 min post-injection (~88% of baseline) and then increased above the baseline (~117%). The oxygen decrease was more rapid and transient than the subsequent oxygen increase. Heroin also decreased oxygen levels in the subcutaneous space (F_17,1547_ = 26.96, p < 0.0001; Fig. 1A); this effect also developed with short latencies, but it was much stronger in amplitude (~60%) and duration than the decrease in brain oxygen.

Heroin also induced a strong hyperthermic response, with significant temperature increases in each recording location (F_33,2013_ = 61.91, 52.22, 20.14 for NAc, muscle and skin, respectively; p < 0.001; Fig. 1B). When analyzed with slow time resolution, the increases were similar in the NAc and muscle (~1.3 °C) but weaker and more delayed in the skin. The increase in NAc temperature was stronger than that in the muscle, resulting in a significant increase in the NAc-muscle differential (F_33,2013_ = 9.69; p < 0.001; Fig. 1C). As shown previously^32^, this effect suggests increased intra-brain heat production due to metabolic brain activation, but it was relatively weak in strength, short in duration and it appeared with a ~10-min latency after heroin injection. In contrast, the skin-muscle differential decreased rapidly, was stronger, and lasted for a longer time after heroin injection (F_33, 2013_ = 18.55; p < 0.001; Fig. 1C). This parameter provides a reliable measure for the tone of skin blood vessels and its decrease suggests powerful peripheral vasoconstriction, which appears to be the primary contributor to heroin-induced brain hyperthermia. Heroin also decreased locomotor activity; this effect was maintained for ~20 min post-injection and was followed by a slight increase in locomotion (Fig. 1D).

More precise dynamics of rapid changes in oxygen and temperature parameters were obtained when the data were analyzed with high, 10-s temporal resolution. As can be seen in Fig. 1E, the drop in NAc oxygen was rapid, but this effect had a definite, ~40–50 s latency and reached nadir at ~130 s from the injection onset (F_23,2783_ = 20.20, p < 0.001). Then oxygen levels began to increase, reaching maximum levels at ~5 min, slowly returning to baseline thereafter. In contrast, oxygen levels in the subcutaneous space decreased with shorter latencies (~30 s) but reached nadir at a later time (~180 s), slowly returning toward baseline thereafter. More precise relationships between heroin-induced changes in oxygen levels in the NAc and subcutaneous space were examined by using time-dependent correlation analysis (Fig. 1H), showing how the changes in one parameter (NAc oxygen) are related to changes in another parameter (subcutaneous oxygen). As can be seen, the latency to oxygen decrease was shorter in the subcutaneous space and the decrease in this location was more rapid and stronger than in brain tissue; both concentration curves tightly correlated from ~30 to 100 s from the onset of heroin injection (r = 0.99). Then, the changes in brain and subcutaneous oxygen levels diverged, as brain oxygen began to increase (~100 s) but subcutaneous levels continued to fall until reaching a plateau (~180 s) and slowly increasing thereafter.

High-resolution analysis also revealed more rapid dynamics of heroin-induced temperature changes (Fig. 1F,G). In this case, skin temperature and skin-muscle differential showed the fastest dynamics, with decreases within the injection duration, but temperature changes in the NAc and muscle were more gradual. NAc temperature and NAc-muscle differentials began to increase after the injection, showing a weak, injection-related increase followed by a larger heroin-induced increase.

### Both naloxone-HCl and naloxone-MET fully block decreases in NAc oxygen induced by iv heroin at a self-administering dose

Next, we examined how each opioid antagonist affects heroin-induced NAc responses. In the first three experiments we examined the effects of sc injections of naloxone-HCl (0.2 mg/kg), naloxone-MET (2.0 mg/kg), and saline on heroin-induced changes in NAc oxygen levels. To establish the link between oxygen decrease and respiratory depression, we also examined how naloxone-MET (2.0 mg/kg) affects heroin-induced oxygen responses in the subcutaneous space. Because of variability in baseline oxygen levels and long duration of heroin responses, the data are shown as relative, percent change vs. pre-injection baseline (=100%) for 60 min following heroin injections. To better represent the initial changes in heroin responses, Supplemental Fig. S1 shows the same data analyzed with high, 10-s time resolution of the initial 10 min post-injection in μM of oxygen.

**Figure 2:**
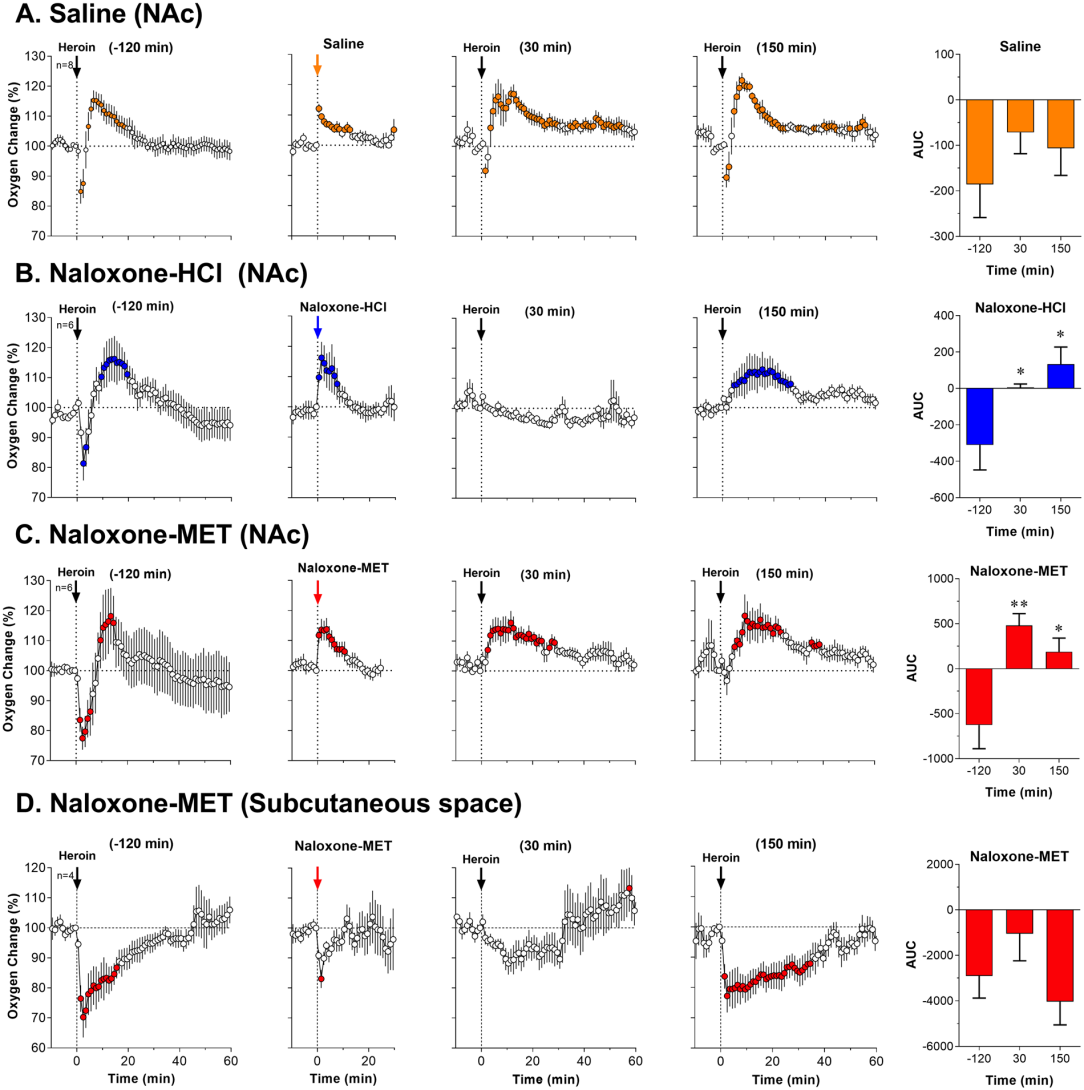
Mean (±SEM) changes in heroin-induced oxygen responses in the NAc (**A**–**C**) and subcutaneous space (**D**) following sc injections of saline (A), naloxone-HCl (**B**; 0.2 mg/kg), and naloxone-MET (**C**,**D**; 2 mg/kg) in freely moving rats. Values significantly different from baseline are shown as filled symbols; n indicates the number of averaged tests. Vertical dotted lines with black arrows show the moments of iv heroin injections and horizontal doted lines show baselines. Right panels with bar graphs show the strength of immediate changes in NAc oxygen calculated as the area under the curve for the time interval of oxygen decrease induced by the initial heroin injection. Negative values represent oxygen decrease and positive values represent oxygen **i**ncrease. Asterisks show significant differences vs. the first heroin injection before administration of each testing drug (*p < 0.05 and **p < 0.01). Second graphs in each row show NAc oxygen responses following sc injection of testing drugs (saline and two naloxone analogs).

As shown in Fig. 2A, heroin induced biphasic NAc oxygen responses following each of the three injections (F_7,637_ = 5.26, 3.65, 6.35, respectively; p < 0.001). In each case, oxygen levels rapidly decreased within a couple of minutes following heroin injection, but then increased above baseline. No significant changes in the strength of heroin-induced NAc oxygen decrease were found when the area under the curve was analyzed (see orange bar graphs on the right side of Fig. 2A).

In contrast to the relative stability of heroin-induced oxygen responses seen after saline injection, these responses were fully blocked when the second heroin injection was delivered 30 min after naloxone-HCl (Fig. 2B; F_5,455_ = 1.00, p = 0.48). While the positive phase of the heroin response was restored when heroin was injected 150 min after naloxone (F_5,455_ = 5.46, p < 0.001), the initial negative phase was still absent. Blockade of NAc oxygen decrease induced by naloxone-HCl was significant when data were analyzed as an area under the curve (see blue bar graphs on the right side of Fig. 2B). In contrast to a clear drop in oxygen following the first injection, this drop was absent for each of the two subsequent heroin injections; the difference vs. pre-naloxone heroin injection was significant in each case (t = 2.28 and 2.67 for the first and second post-naloxone-HCl injections vs. pre-naloxone injection; both p < 0.05).

Similar to naloxone-HCl, administration of naloxone-MET altered the heroin-induced NAc oxygen responses, but the pattern of changes was different (Fig. 2C). When heroin was delivered 30 min after naloxone-MET, it induced only increases in NAc oxygen (F_5,490_ = 2.27, p < 0.001). The decrease in oxygen was partially restored but oxygen levels increased following the second post-naloxone-MET injection (+150 min; F_4,455_ = 5.46, p < 0.001). Significant between-group differences were also detected by analyzing the areas under the curves (see red bar graphs on the right panel in Fig. 2C). The first heroin injection that preceded naloxone-MET decreased NAc oxygen levels for ~7 min, but oxygen levels only increased within this time interval following naloxone-MET injection (t = 3.74, p < 0.01). The initial negative phase of heroin-induced oxygen response was partially restored when heroin was injected at 150 min (t = 2.65, p < 0.05), but the mean change within this time frame was an increase. Similar to our previous experiments^22^, sc injection of saline, naloxone-HCl and naloxone-MET all induced similar NAc oxygen increases (F_7,217_ = 3.56; F_5,165_ = 6.21 and F_5,125_ = 36.74, respectively; p < 0.01) that can be attributed to the stressful nature of the subcutaneous injection procedure.

Although naloxone-MET fully blocked the initial decreases in NAc oxygen levels, suggesting blockade of respiratory depression, it was unable to block the second, increasing phase of the heroin response. To confirm the blockade of respiratory depression and understand the possible mechanisms underlying brain oxygen increases, we examined how naloxone-MET affects heroin-induced oxygen changes in the subcutaneous space (Fig. 2D).

In this experiment we found that the initial heroin injection induced a strong (~75% of baseline) and relatively prolonged (~40 min) decrease in subcutaneous oxygen levels (F_3,273_ = 5.24, p < 0.001) that greatly exceeds the decrease observed in the NAc. When heroin was delivered 30 min after naloxone-MET, this rapid and strong drop in subcutaneous oxygen was absent. Oxygen levels in this case slightly decreased, but the change was slow, weak, and statistically not significant. However, the heroin-induced decrease in oxygen reappeared again at a similar strength when the next heroin injections was administered,150 min after naloxone-MET (F_3,273_ = 4.68; p < 0.001). In contrast to the transient increases in NAc oxygen elicited by all sc injections, oxygen levels in the subcutaneous space showed a weak, transient decrease immediately after the injection.

### Naloxone-HCl and naloxone-MET have distinct effects on heroin-induced changes in brain temperature

**Figure 3:**
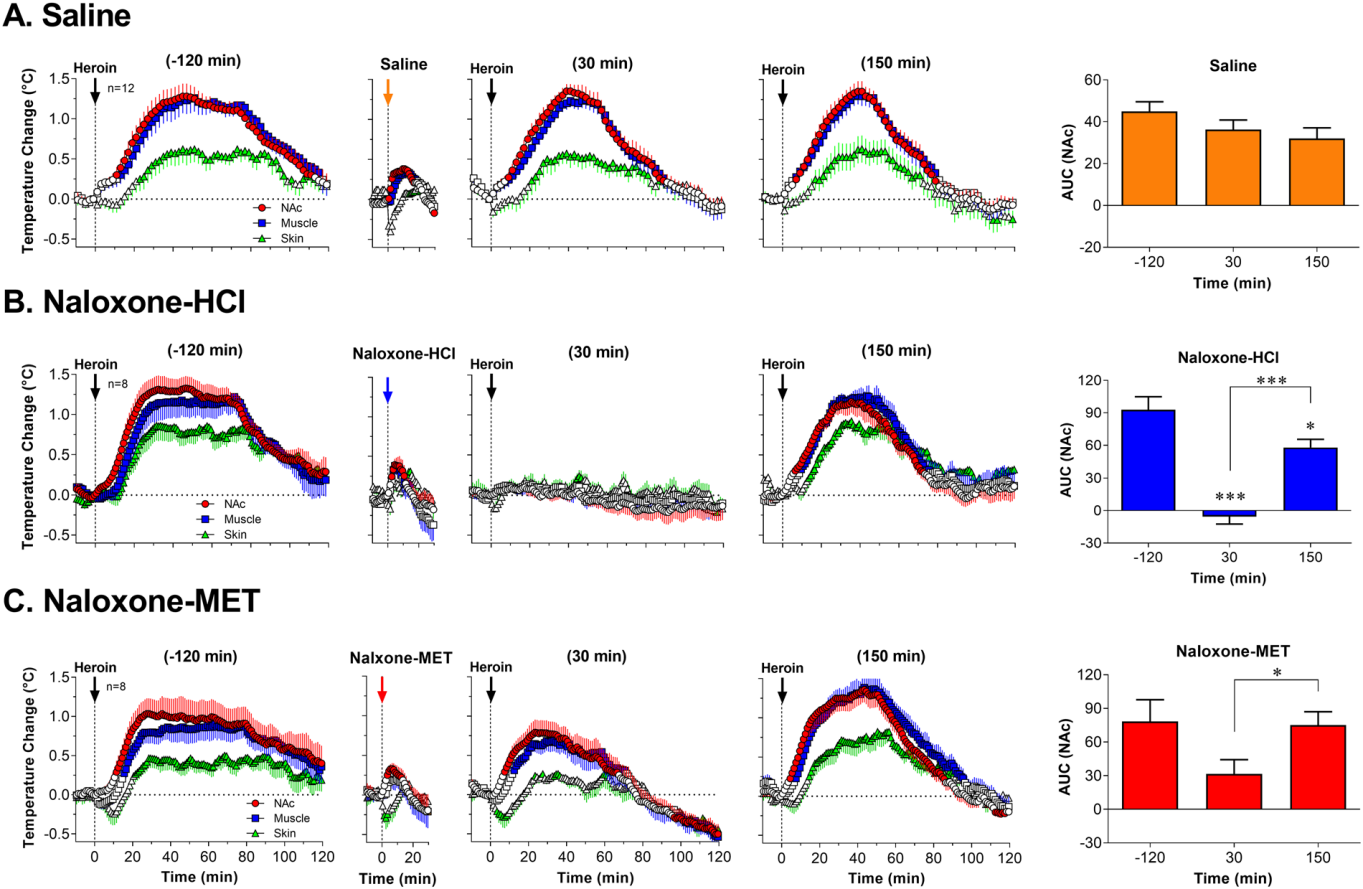
Mean (±SEM) changes in heroin-induced temperature responses in the NAc, temporal muscle and skin following sc injections of saline (**A**, control), naloxone-HCl (**B**, 0.2 mg/kg), and naloxone-MET (**C**, 2 mg/kg). Values significantly different from baseline are shown as filled symbols; n indicates the number of averaged tests. Vertical dotted lines with arrows show the moment of injections and horizontal doted lines show baselines. Right panels with bar graphs show the strength of NAc temperature changes calculated as the area under the curve for 100 min post-injection (interval for NAc temperature increase in control); the values represent averaged temperature increase induced by heroin before and after the administration of each testing drug. Asterisks show significant differences vs. the first heroin injection before administration of each testing drug. Second graphs in each row show temperature responses following sc injection of testing drugs (saline and two naloxone analogs).

Similar to changes in NAc oxygen, heroin-induced temperature responses remained relatively stable following repeated injections (Fig. 3A). In each case, brain and muscle temperature monophasically increased and skin temperature showed a biphasic response, with a slight decrease followed by a large increase. In the NAc, the strength of heroin-induced hyperthermic response as assessed by the area under the curve showed a slight within-session decrease (see orange bars in Fig. 3A). This decrease, however, was not significant.

Naloxone-HCl fully blocked brain hyperthermic response following the second heroin injection (+30 min after naloxone; F_7,840_ = 1.44, NS) and the response was almost fully restored when the next heroin injection was done 150 min after naloxone-HCl (F_7,840_ = 16.32, p < 0.001; Fig. 3B). Full disappearance of heroin-induced brain hyperthermic response was confirmed by analysis of the area under the curve (t = 7.05, p < 0.001; see blue bars in Fig. 3B).

In contrast to naloxone-HCl, naloxone-MET only slightly attenuated heroin-induced temperature responses (F_7,847_ = 18.31 vs. 10.84 for the NAc; Fig. 3C). The increases in brain and body temperature at the first post-treatment heroin injection were slightly weaker but the hyperthermic responses fully restored following the next injection delivered at 150 min after naloxone-MET (F_7,847_ = 54.94; p < 0.001).

Similar to our data with oxygen, sc injections of both opioid antagonists and saline induced weak increases in brain and muscle temperature coupled with transient decreases in skin temperature (Fig. 3A–C). These changes were similar independently of the injected compound and, as shown previously^32^, they were typical to any other salient stimuli.

### Direct assessments of BBB permeability of naloxone-HCl and naloxone-MET

A full blockade of heroin-induced NAc oxygen decreases by naloxone-MET suggests the critical role of peripheral ORs in triggering this effect. However, a possibility still exists that certain amounts of naloxone-MET can cross the BBB and induce this blocking effect by acting directly in the CNS. Due to the lack of objective data demonstrating the BBB permeability of naloxone-MET, we used LC-MS to examine the brain levels of naloxone and methyl-naloxone, the *in vivo* form of naloxone-MET, after sc injections of naloxone-HCl and naloxone-MET.

Since we expected a limited BBB permeability to naloxone-MET, we first examined brain levels of naloxone and methyl-naloxone 30 min after sc injections of naloxone-HCl and naloxone-MET at a higher, 4.3 μmol/kg dose (1.7 mg/kg of naloxone-HCl and 2.0 mg/kg of naloxone-MET). In the second experiment, we injected both naloxone analogs at lower equimolar doses (0.5 μmol/kg; 0.2 mg/kg for naloxone-HCl and 0.24 mg/kg for naloxone-MET). In addition to brain samples, we also measured concentrations of the same substances in kidney and liver.

As shown in Table 1, naloxone-HCl and naloxone-MET injected at higher equimolar doses resulted in the appearance of detectable amounts of methyl-naloxone and naloxone in brain tissue with a ~1:40 concentration ratio. Thus, methyl-naloxone very poorly crosses the BBB. Surprisingly, brains of rats injected with the larger dose of naloxone-MET were found to contain detectable amounts of naloxone. However, naloxone was either not found or found in very low (trace) concentrations in both peripheral organs. With the smaller dose of naloxone-MET, there was no naloxone or methyl-naloxone detected in brain samples.

**Table 1:** Concentrations of methyl-naloxone and naloxone detected in the brain, liver, and kidney after sc injections of naloxone-MET and naloxone-HCl.

Structure	Group	Naloxone	Methyl-Naloxone
Brain	Saline	nd	nd
	Naloxone 4.3 µmol/kg	216.5 ± 19.7	nd
	Naloxone-MET 4.3 µmol/kg	2.3 ± 0.2*	5.3 ± 2.1
	Naloxone 0.5 µmol/kg	32.0 ± 5.4	nd
	Naloxone-MET 0.5 µmol/kg	nd	nd
Kidney	Saline	nd	nd
	Naloxone 4.3 µmol/kg	704.7 ± 216.2	nd
	Naloxone-MET 4.3 µmol/kg	1.0 ± 0.6*	3135.0 ± 841.0
	Naloxone 0.5 µmol/kg	37.9 ± 13.7	nd
	Naloxone-MET 0.5 µmol/kg	nd	579.9 ± 266.2
Liver	Saline	nd	nd
	Naloxone 4.3 µmol/kg	85 ± 4.6	nd
	Naloxone-MET 4.3 µmol/kg	nd	4775.7 ± 571.3
	Naloxone 0.5 µmol/kg	2.5 ± 2.2*	nd
	Naloxone-MET 0.5 µmol/kg	nd	447.9 ± 105.3

Data represents mean(±SD) and are shown in ng/mL. Data for brain were obtained in 4–6 samples and data for peripheral organs obtained in 2–3 samples. nd = not detected; *detectable amounts of the compound but are below the level of quantification.

### Naloxone-MET still potently attenuates heroin-induced NAc oxygen decrease at low doses at which the drug is undetectable in brain tissue

**Figure 4:**
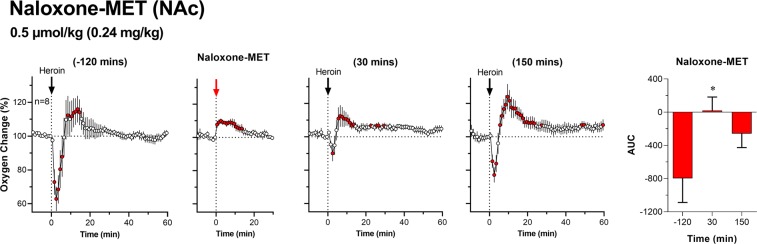
Mean (±SEM) changes in heroin-induced NAc oxygen responses following sc injections of naloxone-MET (0.24 mg/kg) in freely moving rats. Values significantly different from baseline are shown as filled symbols; n indicates the number of averaged tests. Vertical dotted lines with black arrows show the moments of iv heroin injections and horizontal doted lines show baselines. Right panel with bar graph show the strength of immediate changes in NAc oxygen calculated as the area under the curve (interval used was determined by time of decrease of first heroin injection within session); the values represent oxygen decrease (change below 100%) or increase (above 100%) induced by heroin before and after the administration of naloxone-MET. Asterisks show significant differences vs. the first heroin injection before administration of testing drug. Second graph show NAc oxygen response following sc injection of naloxone-MET.

Since our LC-MS experiment revealed no detectable amounts of naloxone or methyl-naloxone after the injection of naloxone-MET at a low dose, we conducted an additional experiment, in which we examined whether heroin-induced NAc oxygen responses (0.1 mg/kg) would be affected by sc administration of naloxone-MET at a lower dose (0.24 mg/kg), the molar equivalent to naloxone HCl at 0.2 mg/kg. As shown in Fig. 4, naloxone-MET at this dose altered heroin-induced NAc oxygen responses differently than at the higher dose. When heroin was delivered 30 min after naloxone-MET, it induced a biphasic response (F_7,637_ = 1.91, p < 0.05), but the first decreasing phase was greatly attenuated. Oxygen decrease was almost fully restored following the second post-naloxone-MET heroin injection (F_7,637_ = 6.68; p < 0.001). Strong attenuation of the heroin-induced oxygen response was evident when analyzing the area under the curve (see red bar graphs on the right panels in Fig. 4). Compared to ~6-min oxygen decrease induced by heroin before naloxone-MET, the decrease became much shorter and weaker following the first post-treatment heroin injection. The area under the curve for the first post naloxone-MET injection was close to zero, due to the weaker oxygen decrease and sooner increase than in the original heroin injection. The area under the curve for the second post-naloxone-MET injection was slightly weaker than this initial one, but the difference was not significant.

**Figure 5:**
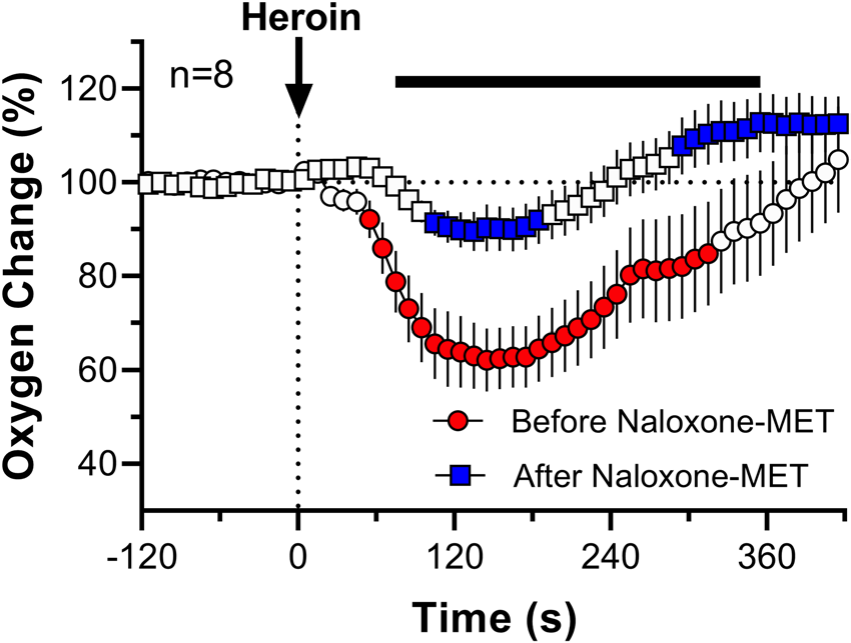
Rapid changes in NAc oxygen levels in rats induced by iv heroin, shown before and after naloxone-MET administration. Data are shown as the mean±SEM in percent vs. baseline ( = 100%). Filled symbols represent values significantly different from pre-injection baseline. Before naloxone-MET injection was 120 min prior to naloxone-MET injection. After naloxone-MET injection was 30 min after naloxone-MET injection. Black horizontal line shows the time interval, during which there were significant differences between the two curves.

In addition to strong attenuation of heroin-induced brain oxygen decrease, naloxone-MET also increased the onset latency of the heroin response (Fig. 5). In contrast to the more rapid oxygen decrease (50–60 s) seen before antagonist treatment, this change occurred with significant latency (100–110 s) when heroin was delivered 30 min after naloxone-MET.

## Discussion

This study’s aim was to examine the possible role of peripheral ORs in triggering respiratory depression and subsequent brain hypoxia induced by opioid drugs. While it is usually believed that these effects result exclusively from the direct interaction of opioid drugs and their metabolites with centrally located ORs, ORs are also abundantly expressed on afferent terminals of visceral sensory nerves innervating blood vessels and internal organs. Therefore, these receptors can be affected by opioids before reaching central substrates and at higher concentrations than those occurring in brain tissue. This study was designed to explore this mechanism with respect to heroin, a prototypical opioid drug of abuse, used at low, behaviorally-relevant doses. In contrast to most studies focused on ventilation aspects of respiration or changes in O_2_ in peripheral blood, the use of oxygen sensors coupled with high-speed amperometry allowed us to directly monitor heroin-induced changes in brain oxygen levels and quantitatively assess brain hypoxia—the functional output of respiratory activity and a clinically relevant parameter.

As a tool for assessing the role of peripheral ORs, we employed an antagonist strategy and examined how heroin-induced NAc oxygen responses are affected by two naloxone analogs—one, which easily crosses the BBB and another, which has no or limited penetration of the BBB. Multiple studies using this strategy hold the belief that naloxone-MET cannot cross the BBB^16–20^ and this was our assumption when we initiated this study. While our first experiment revealed that naloxone-MET at a relatively low dose fully blocks heroin-induced decreases in NAc oxygen, supporting the critical role of peripheral ORs, this conclusion can be valid only if naloxone-MET cannot cross the BBB and affect central ORs. Our second experiment was designed to verify this assumption, using highly sensitive LC-MS measurements of naloxone and methyl-naloxone in brain tissue. Finally, based on the results of these measurements, we conducted the last experiment, in which we examined how the hypoxic effects of iv heroin are affected by naloxone-MET at a much lower dose (0.5 μmol/kg or 0.24 mg/kg) that did not result in any detectable levels of methyl-naloxone in the brain.

This study produced two major findings. First, we provided the first objective analysis of BBB permeability of naloxone-HCl and naloxone-MET, which has major implications for past and future research. Second, we confirmed that interaction of opioids with peripheral ORs plays an important role in triggering brain hypoxia induced by heroin at low, human-relevant doses.

*Respiratory depression as a cause of NAc oxygen decrease induced by heroin*. Consistent with our previous studies^27^, iv heroin induced a biphasic down-up change in NAc oxygen levels. In the subcutaneous space—a densely-vascularized but metabolically inactive area—heroin induced a monophasic oxygen decrease, which was much stronger and more prolonged than in the brain. The initial oxygen decrease in both locations was rapid and tightly correlated, suggesting respiratory depression and subsequent decreases in blood oxygen levels as its cause. However, heroin engages an opposing mechanism that acts to increase brain oxygen levels, causing heroin-induced hypoxia to be weaker in the brain than in the periphery and determining rebound-like brain oxygen increases. As shown previously^22^, brain oxygen levels can increase due to neural activation with subsequent dilation of cerebral vessels and enhanced cerebral blood flow independently of changes in its blood levels. While cerebral vasodilation can occur due to neural activation via a neuro-vascular coupling mechanism^33^ and due to post-hypoxia accumulation of CO_2_, a strong vasodilator^34,35^, it also results from redistribution of arterial blood from the periphery to the brain due to skin vasoconstriction confirmed in our thermorecording experiment. While oxygen decreases resulting from respiratory depression are dose-dependent, being responsible for robust hypoxia following high-dose heroin exposure^27^, the opposing vascular effect is more tonic, weaker, and is limited by the natural propensity of cerebral vessels to dilate.

*Naloxone-MET potently inhibits heroin-induced NAc oxygen decreases*. Consistent with the high affinity of naloxone-HCl to ORs, this drug at a relatively small dose (0.2 mg/kg or 0.5 μmol/kg) fully blocked both oxygen and temperature responses induced by heroin. While heroin-induced hyperthermia reappeared again at the same strength when heroin was injected 2.5 hours after naloxone-HCl, the decreasing phase of NAc oxygen response was still absent at this time. Thus, heroin-induced respiratory depression is more sensitive to full blockade of ORs than other physiological effects, particularly drug-induced changes in brain temperature and vascular tone.

Naloxone-MET at a higher dose (2.0 mg/kg or 4.3 μmol/kg) fully blocked heroin-induced NAc oxygen decreases, pointing at the role of peripheral ORs in triggering this centrally-mediated effect of heroin. However, blockade of peripheral ORs had minimal inhibitory effects on heroin-induced temperature responses (+30 min) and it did not affect heroin-induced brain oxygen increases, indicating their mediation via central ORs. Additional support for the role of peripheral ORs in triggering heroin-induced respiratory depression was obtained in experiments with subcutaneous oxygen measurements. While heroin injected prior to naloxone-MET rapidly and strongly decreased subcutaneous oxygen levels, this effect was absent when the second heroin injection was delivered 30 min after naloxone-MET. The oxygen decrease was basically restored when the next heroin injection was made 2.5 hours after naloxone-MET. However, heroin after naloxone-MET still induced a small tonic decrease in subcutaneous oxygen, resulting from constriction of skin blood vessels as shown in our thermorecording experiment.

*The problem: BBB permeability of naloxone-MET and its possible metabolic degradation*. Although these findings were impressive, blockade of heroin-induced oxygen decreases by naloxone-MET could still be due to two different factors. First, some amount of this drug can cross the BBB, causing a blockage of central ORs. Second, naloxone-MET sample could be contaminated with naloxone or metabolized into this substance *in vivo*, thus affecting our results. These considerations led us to conduct LC-MS measurements to assess BBB permeability of both naloxone analogs.

These measurements resulted in two major findings. First, approximately 5 ng/mL of methyl-naloxone was found in the brain after 4.3 μmol/kg of naloxone-MET was administered. This is approximately 1/40^th^ of the amount of naloxone found in the brain at an equimolar, 1.704 mg/kg dose (1:40 permeability ratio), but only 1/6^th^ of the experimentally used 0.2 mg/kg dose of naloxone-HCl. Though this is significant, the affinity of naloxone-MET to μ ORs is 10–15 times less than that for naloxone, suggesting the overall influence to be very small. While we conducted a high-quality brain perfusion, we cannot be fully confident that this procedure was able to remove all naloxone-MET contained in cerebral vessels. This inescapable factor could also affect the selectivity ratio obtained in our measurements.

The second unexpected finding was that naloxone was found in rats injected with naloxone-MET at a higher dose. Although the amount was very small (2.3 ng/mL) and it is only ~1/94^th^ of the naloxone found in the brain after injection of naloxone-HCl at the same dose (or 1/14^th^ of the lower experimentally used dose), it is possible to speculate that some of the effects of naloxone-MET at the larger doses could be attributed to naloxone found in the brain. This could be due to two factors: original sample contamination or *in-vivo* metabolism of methyl-naloxone into naloxone. First, upon analyzing the standard naloxone-MET sample obtained from Sigma-Aldrich it was found that it contained ~1% of naloxone. This 1% could possibly be the reason for detecting naloxone within brain tissue. There is also a possibility that methyl-naloxone is converted into naloxone *in vivo*. While the brain tissue contained low but detectable levels of naloxone after the large-dose naloxone-MET injection, only traces of this substance were found in both liver and kidney. While this finding argues against systemic metabolic transformation of naloxone-MET into naloxone, we cannot fully exclude this mechanism for brain tissue.

Our measurements of BBB permeability of naloxone-MET could have significant implications for past and present work with this drug. While our data confirm that naloxone-MET has limited BBB permeability, low but quantifiable amounts of naloxone were found in brain tissue. When naloxone-MET is injected at high doses, the effects seen can be the result of the contribution of centrally acting methyl-naloxone and naloxone. Thus, results from prior studies using naloxone-MET could have been affected by both naloxone analogs, though this contribution appears significant only when using high doses of naloxone-MET.

*Peripheral component of heroin-induced brain hypoxia*. Based on results obtained in our LC-MS study, we conducted an additional experiment using naloxone-MET at a much lower dose (0.24 mg/kg), which did not result in any detectable amounts of brain naloxone or methyl-naloxone. Though our data can be theoretically affected by naloxone, the amount of naloxone in the brain will be equivalent to a 0.0014 mg/kg dose of naloxone-HCl. This amount is well below the detection limits of our measurements and naloxone at this dose is thought to induce no effects.

In this experiment NAc oxygen decreases were strongly attenuated by naloxone-MET. The area under the curve for the decreasing phase of oxygen response was ~7 times smaller than that for the original heroin response. Therefore, this experiment underscores the importance of peripherally located ORs in triggering brain hypoxia, a clinically important effect of opioids. These receptors are expressed on the terminals of visceral sensory nerves that innervate blood vessels, carotid bodies, and internal organs and they are also found in the neuromuscular components of the chest wall and diaphragm (pulmonary C-fiber or lung J receptors)^36,37^. Activation of these receptors by heroin and its active metabolites creates a neural signal that rapidly reaches the brain stem neurons via sensory pathways, and by modulating descending neural signals, it changes respiration. The activation of these receptors by endogenous opioid peptides is mild and possibly insignificant under physiological conditions, but their selective over-activation by highly potent opioid agonists has much stronger physiological effects.

When analyzed with fast time resolution, the contribution of peripheral receptors was even more obvious, demonstrating that the latency of heroin-induced NAc oxygen decrease after naloxone-MET was twice as long as that from a normal, pre-treatment heroin injection. This latency could be attributed to the time it takes for heroin to enter the CNS. The appearance of an oxygen decrease post naloxone-MET could result from the inability of naloxone-MET at this low dose to block the effective pool of peripheral ORs. Although we had to decrease the dose of naloxone-MET because of permeability, the affinity of naloxone-MET to μ-opioid receptors is 10 to 15 times less than naloxone-HCl^28–30^. Thus, the small dose of a weaker antagonist could result in peripheral ORs still being open and triggering hypoxia.

### Conclusions and clinical implications

Respiratory depression is usually viewed as a consequence of the direct actions of opioid drugs on “respiratory neurons” located in the brain stem and medulla^3,38^. While the descending efferent signals from the CNS are the ultimate cause of respiratory depression, our study demonstrates that the blockade of peripheral ORs either blocks or strongly attenuates heroin-induced afferent inputs from the periphery to the CNS, thus significantly diminishing respiratory depression and subsequent brain oxygen decreases. Since heroin in this study was used at relatively low but behaviorally relevant doses, these findings cannot be generalized to high-dose opioid use and overdose, when the direct action of these drugs and its metabolites in the CNS will have larger effects on respiratory depression and subsequent brain hypoxia. Under these conditions, the relative contribution of the peripherally triggered component becomes weaker and this mechanism is overshadowed by the more powerful direct action of opioids on central neurons.

Peripherally acting opioid drugs have recently been promoted for pain relief to avoid central side-effects^39,40^. It is often suggested that these drugs would negate the problem of respiratory depression. Our current data demonstrate that though there would be less respiratory depression and brain hypoxia, these drugs are still able to trigger brain hypoxia, which could be significant at high doses. More experimentation will be needed to better understand the effects of peripheral opioids and the functional role of peripheral opioid receptors.

## Materials and Methods

### Subjects

57 adult male Long-Evans rats (Charles River Laboratories) weighing 460 ± 40 g at the time of surgery were used in this study. Rats were individually housed in a climate-controlled animal colony maintained on a 12–12 light-dark cycle with food and water available ad libitum. All procedures were approved by the NIDA-IRP Animal Care and Use Committee and complied with the Guide for the Care and Use of Laboratory Animals (NIH, Publication 865-23). Maximal care was taken to minimize the number of experimental animals and any possible discomfort or suffering at all stages of the study.

### Overview of the study

In this study, we describe the results of three types of experiments (oxygen electrochemistry, thermorecording, and LCMS measurements). In our electrochemical experiments (n = 24 rats), we examined how naloxone-HCl, naloxone-MET, and saline affect NAc oxygen responses induced by iv heroin in awake, freely moving rats. In two additional electrochemical experiments (n = 7 rats), we examined heroin-induced changes in subcutaneous oxygen levels and the influence of naloxone-MET on such heroin-induced oxygen responses. In the thermorecording experiments (n = 12 rats) conducted with the same protocol we examined how these same treatments affect heroin-induced changes in NAc, temporal muscle, and skin temperatures. Finally, we used LCMS measurements to quantitatively examine how both naloxone analogs enter brain tissue (n = 14 rats).

### Surgical preparations for electrochemical and temperature experiments

Surgical procedures for electrochemical assessment of oxygen have been described in detail elsewhere^22^. Briefly, under general anesthesia (Equithesin, a mixture of sodium pentobarbital and chloral hydrate), rats were chronically implanted with a Pt-Ir oxygen sensor (Model 7002-02; Pinnacle Technology, Inc., Lawrence, KS, USA) into the NAc shell and the subcutaneous space. Target coordinates for the right NAc were: AP + 1.2 mm, ML ± 0.8 mm, and DV + 7.6 mm from the skull surface, according to coordinates of the rat brain atlas^41^. The subcutaneous sensor was implanted under the skin in the medio-frontal area of the rat’s head. The sensor was secured with dental acrylic to three stainless steel screws threaded into the skull. During the same surgery, rats were also implanted with a chronic jugular catheter, which ran subcutaneously to the head mount. Rats were allowed a minimum of 5 days of post-operative recovery and at least 3 daily habituation sessions (~6 h each) to the recording environment. Jugular catheters were flushed daily with 0.2 ml heparinized saline to maintain patency.

Surgical procedures for thermorecording experiments have been described in detail elsewhere^42,43^. Briefly, under the same anesthesia protocol, rats were implanted with a jugular catheter and three copper-constantan thermocouple electrodes in the NAc shell, temporal muscle, and subcutaneously along the nasal ridge with the tip ~15 mm anterior to bregma. Probes were secured with dental acrylic to three screws threaded into the skull. Rats were given a minimum of 5 days to recover post operation. Rats were also allowed at least 3 habituation sessions (~6 h each) to the recording environment prior to experimentation. Jugular catheters were flushed daily with 0.2 ml heparinized saline to maintain patency.

### Electrochemical detection of oxygen

For *in vivo* oxygen detection we used Pinnacle oxygen sensors coupled with high-speed amperometry. These sensors were prepared from 180 μm Pt-Ir wire and had a sensing area of 0.025 mm^2^ at the tip. The active electrode was incorporated with an integrated Ag/AgCl reference electrode. Dissolved oxygen is reduced on the active surface of these sensors, held at a stable potential of −0.6 V vs. the reference electrode, producing an amperometric current. The current from the sensor was then transmitted via a potentiostat (Model 3104, Pinnacle Technology) to the computer and recorded at 1-s intervals, using PAL software (Pinnacle Technology).

Oxygen sensors were calibrated at 37 °C by the manufacturer (Pinnacle Technology) according to a standard protocol described elsewhere^44^. The sensors produced linear current changes with increases in oxygen concentrations within a wide range of brain oxygen concentrations (0–40 μM). Substrate sensitivity of oxygen sensors varied from 0.67 to 1.41 nA/1 μM (mean = 0.84 nA/1 μM). Oxygen sensors were also tested by the manufacturer for their selectivity toward electroactive substances such as dopamine (0.4 μM) and ascorbate (250 μM), none of which had significant effects on current measurements.

### Experimental procedures for electrochemical and temperature experiments

At the onset of each experiment, rats were briefly anesthetized (<2 min) with isoflurane. During this time either the electrochemical or temperature sensors were connected to the recording instrument via an electrically shielded flexible cable and a multi-channel electrical swivel. A catheter extension was used to allow for stress and cue-free drug delivery from outside the cage. Testing began approximately 120 min after the temperature or electrochemical sensors were connected to the recording instruments, allowing for baseline currents to stabilize.

In our electrochemical experiments, we examined how four types of treatments (saline = control, naloxone-HCl 0.2 mg/kg, and naloxone-MET 2 mg/kg and 0.24 mg/kg) affect changes in NAc oxygen induced by iv heroin (diacetylmorphine HCl, obtained from NIDA-IRP Pharmacy; 0.1 mg/kg in 0.4 ml saline). Both naloxone analogs were obtained from Sigma-Aldrich. Rats in these experiments received three heroin injections during an ~8-hour recording session. 120 min after the initial heroin injection, rats received a subcutaneous (sc) injection of one of three testing drugs (naloxone-HCl, naloxone-MET, or saline in 0.4 ml volumes). Second and third heroin injections were delivered 30 and 150 min after the injection of each testing drug. At the end of a session, rats were injected with Equithesin (0.6–0.8 ml) to safely disconnect the animals from the recording instruments. Data with naloxone analogs and saline were obtained in four different groups of rats. In two additional electrochemical experiments, we used a similar protocol to examine how heroin (0.1 mg/kg) affects oxygen levels in the subcutaneous space and how naloxone-MET (2 mg/kg) affects heroin-induced changes in subcutaneous oxygen levels.

A similar protocol was maintained in our thermorecording experiments, in which rats received three heroin injections—one before and two at 30 and 150 min after the injection of each testing drug. In these experiments, we also monitored locomotion activity using 4 infrared motion detectors (Med Associates) as previously described^45^. Control data in both experiments (heroin injections after saline) were obtained in separate groups of rats, which were recorded for two daily sessions. Data with both naloxone analogs were obtained in two different groups of rats, with each rat receiving one injection of naloxone-HCl and one injection of naloxone-MET in a counter-balanced manner.

### Mass-spectrometry assessment of brain levels of naloxone and naloxone-MET after their systemic administration

These experiments were conducted in drug-naive rats, which were anesthetized with isoflurane, injected with naloxone HCl, naloxone-MET, or saline, and perfused with room-temperature heparinized saline (30 mL/min for ~15 min, 2000 Units/100 mL). Because of our expectation of limited permeability of naloxone-MET, in the first experiment, both naloxone analogs were used at a 4.3 μmol/kg dose (2.0 mg/kg naloxone-MET and 1.70 mg/kg of naloxone-HCl) to ensure sufficient quantity for detection in brain tissue. In the second measurement experiment, we used low doses of these drugs (0.5 μmol/kg: naloxone-MET 0.235 mg/kg and 0.2 mg/kg of naloxone-HCl) identical to those used in our last oxygen experiment. At the time of initial anesthesia, rats were injected with 1000 units of heparin to prevent blood coagulation that could affect brain levels of measured substances. Perfusion began at 30 min after the injection of tested drugs. Similar to oxygen and temperature experiments, both naloxone analogs were injected subcutaneously in 0.4 ml of saline. Brains were rapidly extracted, cut in half, and frozen on dry ice and later transferred into a freezer at −20 °C. We also took one lobe of liver and left kidney as control peripheral organs.

Brain, liver and kidney samples were homogenized in 2 mL of water per gram of tissue. For extraction, 200 μL of the tissue homogenates were mixed with 800 µL acetonitrile. The samples were then vortexed for 30 min and centrifuged at 14000 rpm for 15 minutes before being stored at −20 °C for 1 hour. Next, the supernatant was transferred and evaporated at 50 °C under nitrogen. Finally, the samples were resuspended in 200 µL of 6% methanol in 0.1% formic acid for mass analysis. Samples were analyzed on a Dionex UltiMate 3000 HPLC coupled to an Orbitrap Velos with a HESI source (Thermo Scientific, San Jose, CA). A Kinetex® 2.6 µm C18 100 Å (100 × 2.1 mm) column from Phenomenex (Torrance, CA) was used for liquid chromatography separation. Liquid chromatography conditions were as follow: column temperature, 30 °C; mobile phase A (aqueous) 0.1% formic acid and mobile phase B (organic) was methanol; flow-rate, 0.3 mL/mi; gradient, from 6% to 100% mobile phase B in 8 min; sample injection volume, 5 μL. ESI–MS parameters were: source voltage = 4 kV; positive ion mode; mass resolution = 60,000. A standard curve was generated for naloxone-HCl and naloxone-MET from 1 to 5000 ng/mL. The limit of quantification was 5 ng/mL and the limit of detection was 1 ng/mL for both naloxone-HCl and naloxone-MET. The retention time for naloxone was 3.55 and for naloxone-MET 3.35 minutes. Standard samples of naloxone-MET with high concentrations showed a mass peak corresponding to naloxone (~1%), suggesting the presence of a very small amount of naloxone in the sample.

### Histological verification of electrode placements

When electrochemical and temperature recordings were completed, rats were deeply anesthetized with isoflurane, decapitated, the brains were extracted, and stored in 10% formalin solution. Later, the brains were sectioned and analyzed for verification of the locations of cerebral implants and tissue damage.

### Data analysis

Electrochemical data was analyzed with slow (1-min) and rapid (10-s) time resolution. Because each individual sensor differed in substrate sensitivity, currents were converted into concentrations according to sensitivity calibrations provided by the manufacturer. Data were then converted into % changes in concentration of oxygen. Values from one minute prior to injection were averaged and used to set the 100% baseline. One-way repeated measure ANOVAs (followed by Fisher LSD post-hoc tests) were used to evaluate statistical significance of drug/antagonist-induced oxygen changes. Decreases in oxygen were also represented as the area under the curve (AUC) calculated for the time interval of oxygen decrease during the first (control) heroin injection in each treatment group. To determine this parameter, we subtracted 100 from each 10-s oxygen percent value, summated all the points within a time interval of mean decrease in pre-treatment control and then averaged these values to determine the means and standard errors for the initial, pre-treatment and two post-treatment heroin injections. These mean values were statistically compared using Student’s t-test. Similar procedures were used to analyze temperature data. In addition to drug-induced temperature changes in the NAc, temporal muscle and skin, we also determined NAc-muscle and skin-muscle differentials, which reflect changes in intra-brain heat production due to metabolic activation/inhibition and in peripheral heat loss due to changes in skin vascular tone (vasoconstriction/vasodilation). Changes in temperature parameters are shown as changes relative to pre-injection baseline (set to 0 °C).

## Supplementary information

Supplementary Information.

## Data Availability

Raw data and the results of their primary analyses are available on request.
