# Supplementary material for: The Role of Peripheral Opioid Receptors in Triggering Heroin-induced Brain Hypoxia
Source: Sci Rep. 2020 Jan 21;10:833. doi: 10.1038/s41598-020-57768-3 (PMC6972941; doi:10.1038/s41598-020-57768-3)
Supplement: Supplementary file 1 — Supplementary Information. [file 41598_2020_57768_MOESM1_ESM.pdf]

Supplementary information to:

The role of peripheral opioid receptors in triggering heroin-induced brain hypoxia

David Perekopskiy, Anum Afzal, Shelley N. Jackson, Ludovic Muller, Amina S. Woods, Eugene A. Kiyatkin*

Behavioral Neuroscience Branch, National Institute on Drug Abuse – Intramural Research Program, National Institutes of Health, DHHS, 333 Cassell Drive, Baltimore, MD 21224, USA

Address correspondence to Eugene A. Kiyatkin at ekiyatki@intra.nida.nih.gov

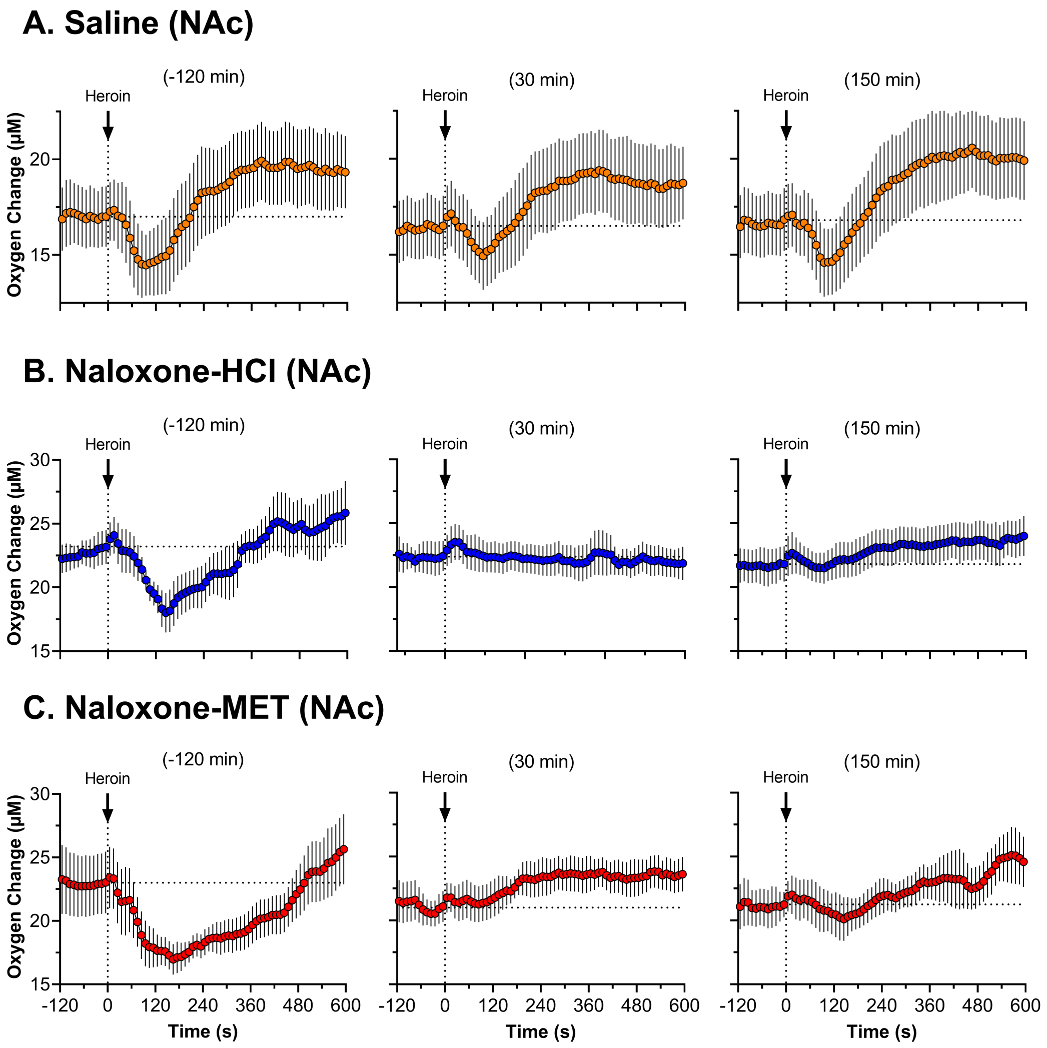

Figure S1. Mean (±SEM) changes in heroin-induced oxygen responses in the NAc following sc injections of saline (A), naloxone-HCl (B, 0.2 mg/kg), and naloxone-MET (C, 2 mg/kg) in freely moving rats. Data are shown as concentration change with 10-s time resolution for the first 10 min after heroin injections. Vertical dotted lines with black arrows (heroin) show the moments of iv heroin injections and horizontal doted lines show baselines.

In contrast to Fig. 2 in the main text, in which data are shown as a relative change with 1-min time resolution for 60 min post-injection, data analyzed using absolute concentration change have higher variability due to differences in basal oxygen levels in each individual rat. Despite larger standard errors, the pattern of heroin-induced NAc oxygen response is virtually identical with both types of data presentation.
